# Research advances in physical activity and insomnia: a bibliometric and thematic evolution analysis

**DOI:** 10.3389/fpsyt.2026.1801508

**Published:** 2026-06-10

**Authors:** Junrong Chen, Shengchao Wang, Yeting Zhang

**Affiliations:** 1 College of Physical Education, Chengdu University, Chengdu, China; 2 College of Computer Science, Chengdu University, Chengdu, China; 3 College of Aviation Physical Education, Civil Aviation Flight University of China, Guanghan, China

**Keywords:** bibliometrics, insomnia, physical activity, research hotspots, visualization

## Abstract

**Background:**

Insomnia affects approximately 16.2% of the global population, constituting a substantial public health burden associated with diverse adverse health outcomes. Although physical activity (PA) represents a modifiable health behavior with demonstrated potential for sleep improvement, a systematic mapping of research hotspots and evolutionary trajectories in this domain remains lacking.

**Methods:**

We systematically retrieved English-language publications from the Web of Science Core Collection (WoSCC) from inception through May 20, 2026. A comprehensive search strategy combining subject headings with free-text keywords yielded 2,224 included records. We utilized the Bibliometrix package in R and VOSviewer software to visualize publication patterns across countries, institutions, journals, and authors; constructed thematic evolution maps; and identified research hotspots and emerging trends through strategic diagram analysis. We retrieved a supplementary Scopus dataset for cross-database validation. Web of Science served as the primary data source for analyses and visualizations, while Scopus verified trend consistency.

**Results:**

The annual growth rate of publications reached 15.12%. The United States (n = 397) and China (n = 462) emerged as the predominant contributing nations. High-impact institutions included Harvard University and the University of California system. Among journals, Sleep Medicine exhibited the highest h-index. Thematic evolution revealed four distinct phases: 2010–2018 focused on metabolic associations and measurement instruments; 2019–2021 shifted toward pandemic-related stress and sleep-emotion comorbidities; 2022–2023 established a “monitoring-intervention-evaluation” closed-loop paradigm; and 2024–2025 entered a “genetic causality-digital intervention” era. Mendelian randomization(MR) and wearable devices have emerged as cutting-edge frontiers. Cross-database validation with Scopus corroborated a high degree of concordance in publication trends and core thematic foci.

**Conclusion:**

The PA-insomnia research field has completed a paradigm shift from metabolic association validation to genetic causal precision, currently positioned at a critical juncture of multidisciplinary integration and digital transformation. Future research priorities might include, developing artificial intelligence-enabled wearable systems for real-time sleep-emotion-metabolism digital twin modeling, and advocating for the integration of multi-component “sleep hygiene plus exercise” interventions into clinical practice guidelines to address escalating sleep health challenges amid global population aging. Cross-database validation with Scopus corroborated a high degree of concordance in publication trends and core thematic foci.

## 1 Introduction

Insomnia is clinically characterized by difficulties in sleep onset, sleep maintenance, or premature morning awakening with subsequent daytime impairment. Epidemiological surveys indicate a global prevalence of approximately 16.2% for general insomnia disorders, with severe manifestations affecting an estimated 7.9% of the population; notably, both prevalence rates demonstrate a progressive age-dependent escalation (1). Chronic insomnia precipitates substantial decrements in quality of life and is robustly associated with a spectrum of adverse health outcomes, including major depressive disorder, generalized anxiety disorder, metabolic syndrome, cardiovascular morbidity, and accelerated cognitive decline, prompting the World Health Organization to designate this condition as an “under-addressed public health challenge” (2, 3). Physical activity (PA) is defined as any bodily movement produced by skeletal muscles that requires energy expenditure, encompassing activities undertaken across leisure, occupation, transport, and domestic domains (4). Concurrently, physical activity (PA) functions as a modifiable lifestyle factor with demonstrated efficacy in enhancing sleep continuity, augmenting slow-wave sleep (SWS) duration, and stabilizing circadian rhythmicity (5–7). In the context of insomnia research, PA interventions are commonly classified into three categories: (i) aerobic exercise (e.g., walking, jogging, cycling), typically performed at moderate intensity (3.0–<6.0 METs) for 150–300 minutes per week or vigorous intensity (≥6.0 METs) for 75–150 minutes per week; (ii) muscle-strengthening activity (e.g., resistance training), involving major muscle groups on ≥2 days per week; and (iii) mind–body exercises (e.g., yoga, Tai Chi), characterized by integrated movement, breath regulation, and meditative attention, typically practiced for 45–90 minutes per session (8–10). The sleep-promoting mechanisms operate through multiple pathways: first, by optimizing sleep architecture via increasing non-rapid eye movement (NREM) sleep and attenuating nocturnal arousals; second, by amplifying melatonin rhythm amplitude and phase stability; and third, by serving as a non-photic zeitgeber capable of modulating core clock gene expression (7). Moreover, accumulating systematic reviews and meta-analyses substantiate the therapeutic utility of exercise interventions as adjuvant treatments for insomnia (11, 12).

Currently, reviews in the PA and insomnia domain have predominantly taken the form of systematic reviews and meta-analyses, which are inherently constrained to examining specific effect sizes of PA interventions on insomnia outcomes rather than providing a comprehensive, systematic mapping of the research landscape. Bibliometric methodology enables quantitative analysis of bibliographic records through mathematical and statistical techniques (13), allowing researchers to extract pertinent information from large-scale literature corpuses, identify research hotspots and evolutionary trends within specific domains, and determine future research priorities (14). This approach has been widely applied in geriatrics, neuroscience, and exercise science (15). However, no studies to date have employed bibliometric visualization tools to examine research hotspots and trends at the intersection of PA and insomnia. Given this knowledge gap, the present study utilizes bibliometric software to analyze the evolutionary trajectory of the PA-insomnia research domain, translating complex developmental processes into intelligible visualizations to elucidate the field’s current state and intellectual structure, synthesize prevailing research hotspots and emerging trends, and provide evidence-based directions for future inquiry.

## 2 Methods

### 2.1 Data sources and search strategy

**Figure 1:**
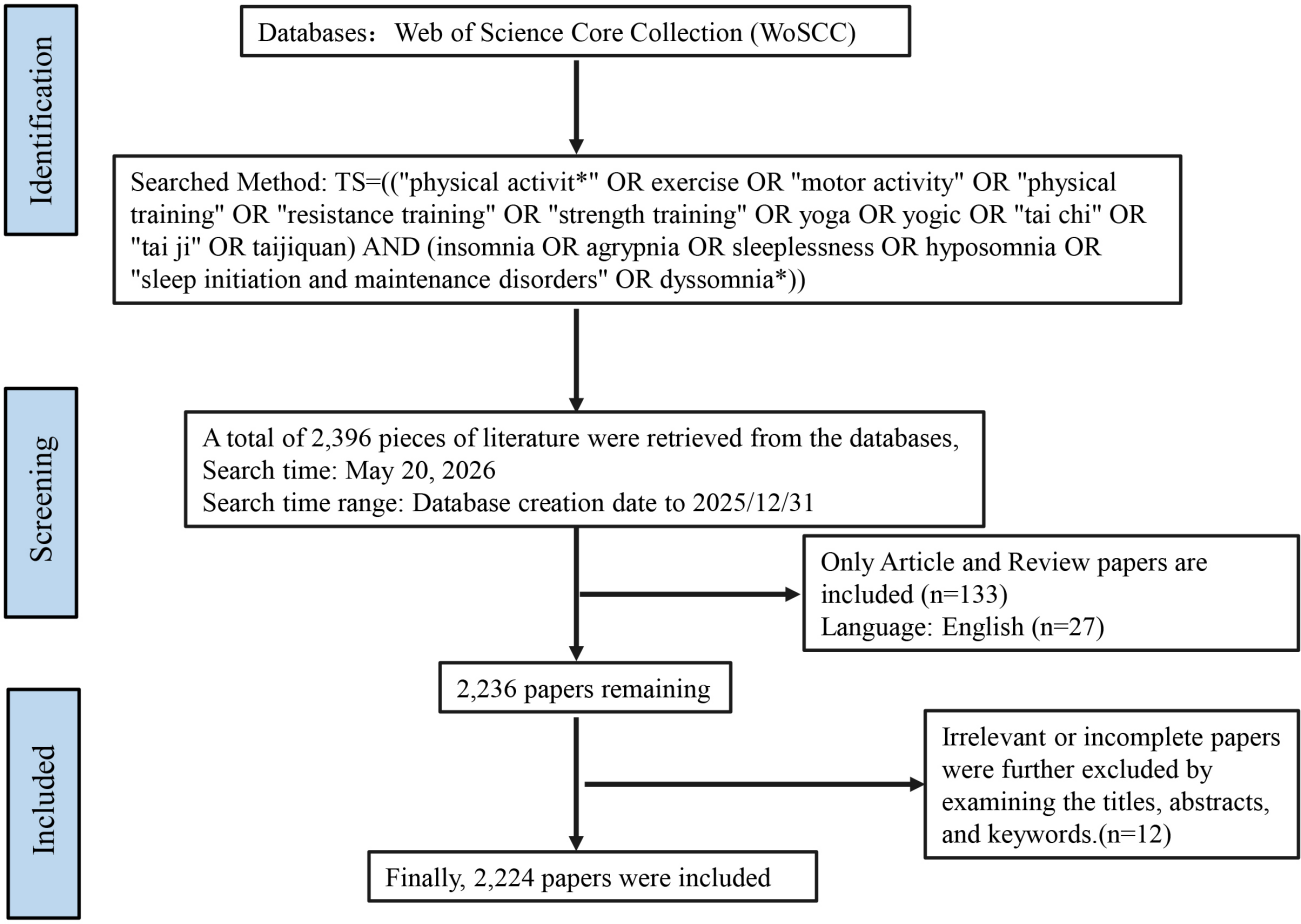
Flow diagram for the screening.

Data for this study were retrieved from two independent databases—the Web of Science (WOS) Core Collection and Scopus—for rigorous bibliometric analysis and cross-database validation. The publication timeframe was restricted to 2010–2025 in both databases. To ensure both comprehensiveness and specificity in literature identification, we employed a hybrid search strategy integrating controlled vocabulary (subject headings) with free-text keywords. This approach facilitated exhaustive coverage of core domain concepts alongside pertinent alternative terminologies. Systematic searches were conducted across Web of Science Core Collection (WoSCC) from database inception through December 31, 2025. The search string applied to WoSCC was formulated as follows: TS=((“physical activit*” OR exercise OR “motor activity” OR “physical training” OR “resistance training” OR “strength training” OR yoga OR yogic OR “tai chi” OR “tai ji” OR taijiquan) AND (insomnia OR agrypnia OR sleeplessness OR hyposomnia OR “sleep initiation and maintenance disorders” OR dyssomnia*)), with document types restricted to Articles and Reviews. WoSCC was selected as the sole data source because it provides complete bibliographic metadata—including cited references, author affiliations, and institutional addresses—essential for co-citation analysis, bibliographic coupling, and collaboration network mapping. The initial search yielded 2,396 records. Two investigators (Chen and Wang) independently conducted title/abstract screening and full-text eligibility assessment using pre-defined exclusion criteria. Inter-rater agreement was calculated using Cohen’s kappa coefficient. Discrepancies were resolved through consensus discussion; a third senior investigator (Zhang) was consulted when consensus could not be reached. Inclusion criteria comprised: (1) English-language publications; (2) research articles or reviews; (3) complete bibliographic metadata (title, authors, abstract, keywords, references, publication year); (4) topical relevance to physical activity and insomnia as determined by both investigators. Exclusion criteria comprised: (1) non-English publications; (2) duplicate records; (3) incomplete bibliographic metadata; (4) topical irrelevance; and (5) non-research publications (editorials, news items, correspondence, and conference abstracts). Ultimately, 2,224 studies met the eligibility criteria and were included in the bibliometric analysis. The literature selection workflow is presented in **Figure 1**.

To ensure the reliability of our findings and mitigate potential biases arising from database-specific characteristics, we retrieved a parallel validation dataset from Scopus using the following conceptually equivalent search strategy: TITLE-ABS-KEY((“physical activit*” OR exercise OR “motor activity” OR “physical training” OR “resistance training” OR “strength training” OR yoga OR yogic OR “tai chi” OR “tai ji” OR taijiquan) AND (insomnia OR agrypnia OR sleeplessness OR hyposomnia OR “sleep initiation and maintenance disorders” OR dyssomnia*)). Ultimately, 4,754 records were included for comparative validation analysis. The literature selection process is illustrated in **Supplementary Figure 1**.

### 2.2 Data analysis

Bibliometric analyses were conducted using R software (version 4.1.2; R Foundation for Statistical Computing, Vienna, Austria) with the bibliometrix package (version 3.0.3; http://www.bibliometrix.org) and VOSviewer (version 1.6.18) for data visualization and network analysis (16, 17) Descriptive bibliometric indicators were calculated to examine publication patterns across documents, countries, institutions, journals, and authors. Specifically, frequency distributions and citation metrics were computed for each category, with the top 20 entities ranked accordingly. Temporal thematic evolution was analyzed over the period 2010–2025. To standardize the keyword corpus, two auxiliary plain text files were constructed: a synonym file to consolidate synonymous terms and an exclusion file to filter duplicate or non-informative keywords (e.g., search terms). Keyword frequency trajectories were tracked longitudinally to identify temporal patterns, and thematic maps were generated for distinct time intervals to illustrate the developmental trajectory of research topics. An integrated analytical framework was adopted to ensure methodological consistency and reproducibility throughout the analysis (17). Default parameters were utilized for all software configurations unless otherwise specified. Analyses were performed using Biblioshiny, a web-based interface for the bibliometrix package, facilitating interactive data visualization and social network mapping. Additionally, VOSviewer (version 1.6.18; developed by Nees Jan van Eck and Ludo Waltman, Centre for Science and Technology Studies, Leiden University; https://www.VOSviewer.com) was employed to construct co-authorship, international collaboration, institutional affiliation, and keyword co-occurrence networks. In these visualizations, node size denotes frequency or citation count, while link thickness represents collaboration intensity. Minimum occurrence thresholds were established at 10 for authors, institutions, journals, and keywords; at 5 for countries; and at 100 for document citations.

## 3 Results

### 3.1 Overview of publications and time trends

**Figure 2:**
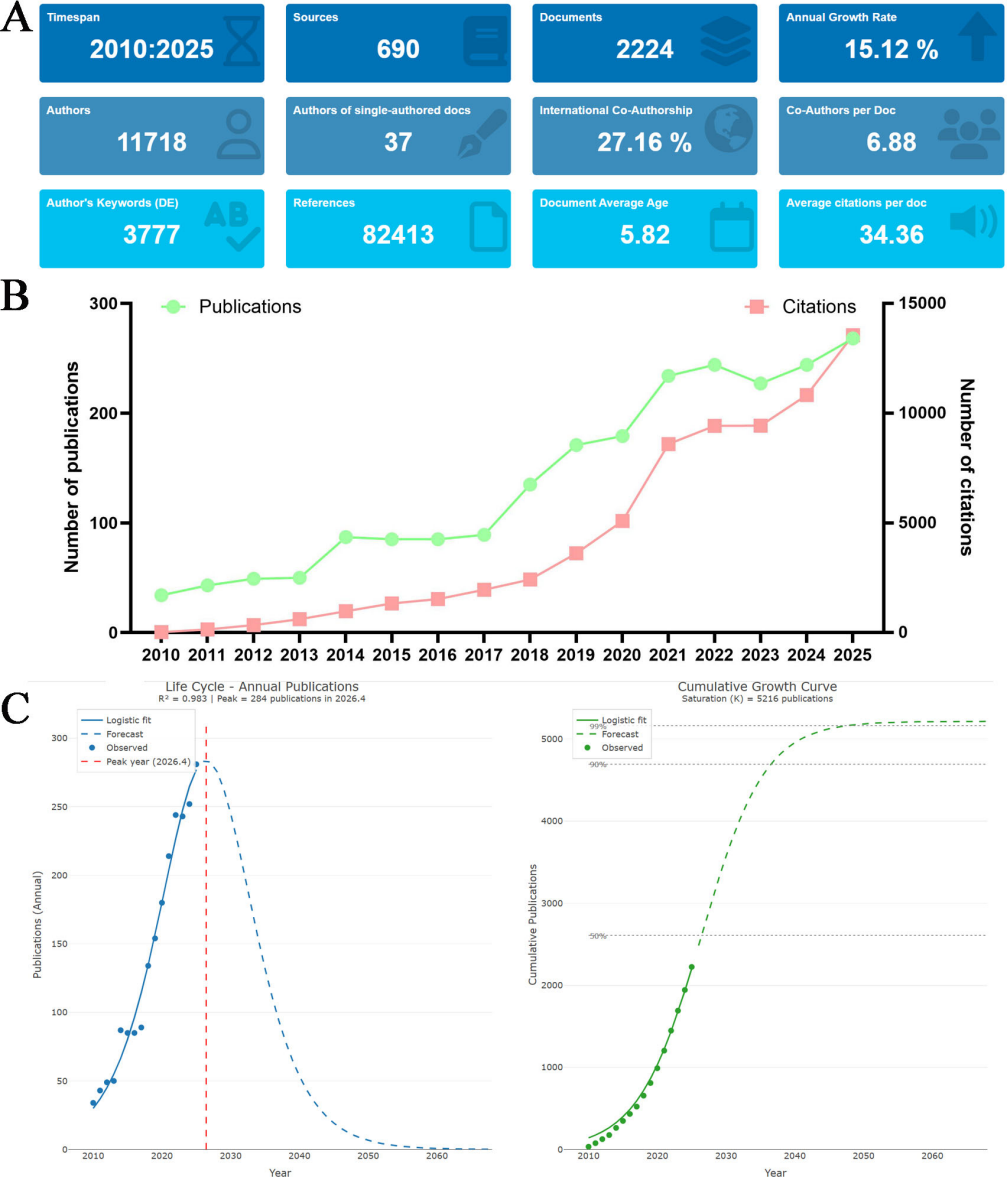
Bibliometric profile and lifecycle analysis of the included literature. **(A)** Descriptive characteristics of publications across countries, institutions, authors, and journals. **(B)** Annual publication trends and cumulative citations, 2010–2025. **(C)** Predictive growth model showing the field’s lifecycle trajectory and saturation point.

Following eligibility screening and assessment, 2,224 publications addressing physical activity and insomnia were ultimately included for bibliometric analysis (**Figure 2A**). These documents were authored by 11,718 researchers affiliated with 2,147 institutions across 67 countries, published in 668 journals, and cited 52,468 references from 5,220 source journals. Between 2010 and 2025, annual publication output demonstrated a modest upward trajectory with an annual growth rate of 15.12%. As illustrated in **Figure 2B**, a conspicuous publication surge occurred in 2014 (n = 87), followed by a sharp escalation post-2017, culminating in a record output of 244 publications in 2024—a trend projected to persist. **Figure 2C** presents predictive modeling indicating that the field will reach its maximum annual productivity of 254 publications by April 2026, at which point cumulative output will approximate 90% of the theoretical saturation limit (estimated at 5,216 total publications). Subsequently, annual publication counts are forecasted to decline progressively while cumulative growth plateaus; by approximately 2032, the field will reach saturation with a residual growth margin of fewer than 500 publications. These patterns suggest the research domain has entered a peaking-decline phase characterized by diminishing returns, whereby continued expansion will prove unsustainable without substantive infusion of novel paradigms or interdisciplinary breakthroughs. Predictive growth modeling was conducted using the logistic growth function implemented in Biblioshiny (Bibliometrix package, version 3.0.3). The model was fitted to annual publication counts from 2010 to 2025 via nonlinear least squares regression: P(t) = K/[1 + exp(−b(t − t_0_))], where K denotes the saturation level (estimated maximum cumulative publications), b the intrinsic growth rate, and t_0_ the inflection point corresponding to the year of peak annual productivity. Parameter estimation employed the Levenberg–Marquardt algorithm. The model demonstrated excellent fit to the observed data (R² = 0.98), yielding a saturation estimate of K = 5,216 total publications and a peak year of Tm = 2026. It should be emphasized that this projection assumes smooth, uninterrupted logistic growth and is inherently sensitive to disruptive events such as paradigm shifts, funding fluctuations, or technological breakthroughs; consequently, the saturation estimate should be interpreted as a theoretical asymptote under current growth parameters rather than a deterministic forecast.

### 3.2 Analysis of countries

**Figure 3:**
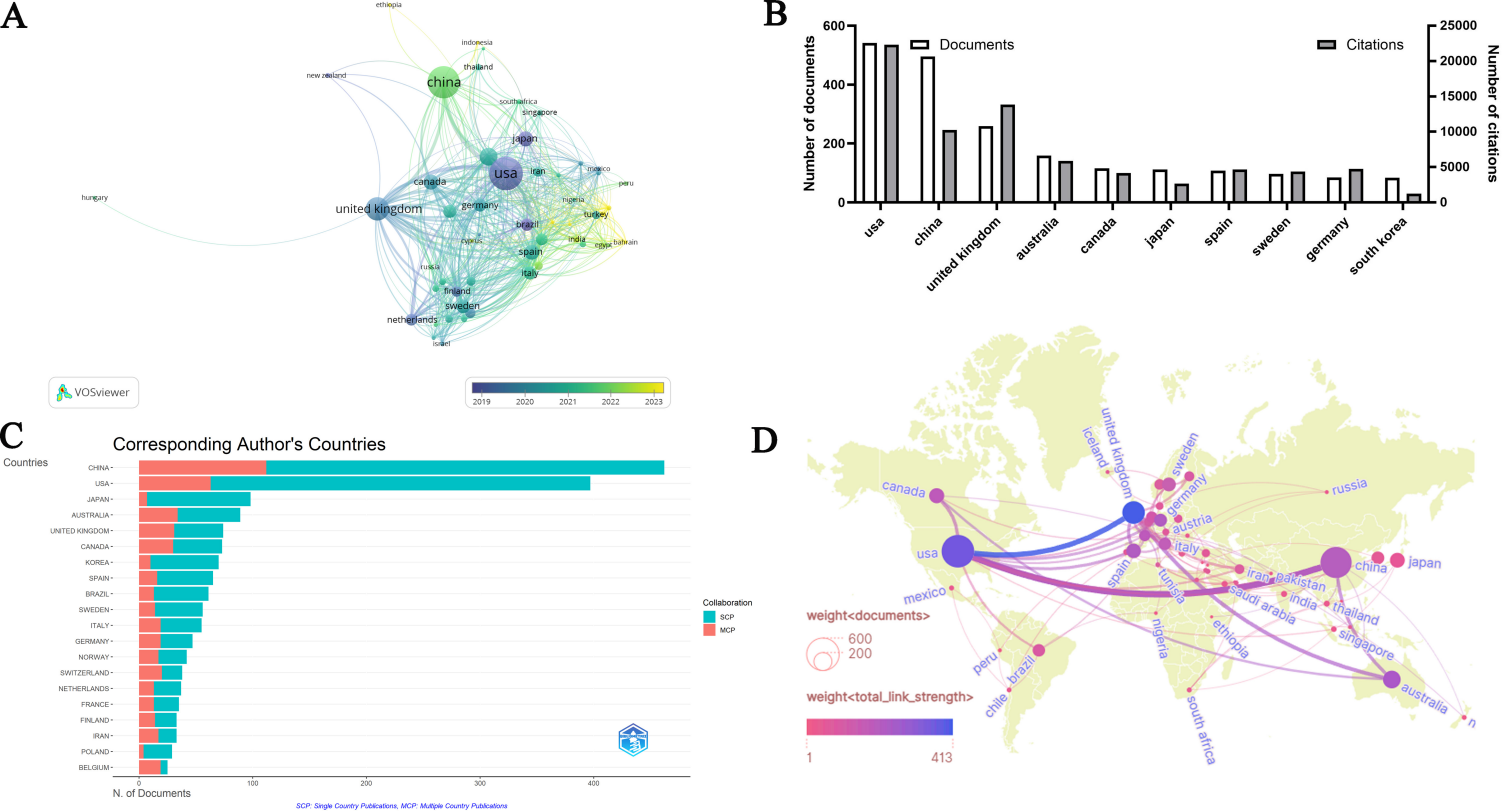
Geographic distribution and international collaboration patterns. **(A)** Network visualization of co-authorship relationships among countries (node size = publication volume; link thickness = collaboration frequency). **(B)** Top 10 contributing countries ranked by total publication output. **(C)** Distribution of articles by corresponding author country, stratified by single-country (SCP) and multiple-country (MCP) publications. **(D)** Geographic mapping of international co-authorship links (total link strength).

A total of 67 countries contributed to physical activity and insomnia research. Based on corresponding author affiliations, China and the United States collectively produced over 800 publications, substantially outpacing all other nations. While 29 countries contributed more than 10 articles each, only 11 nations exceeded 50 publications; conversely, 16 countries were represented by a single article (**Figure 3A**). China emerged as the most prolific contributor with 462 articles (22.3% of the total corpus), comprising 350 single-country publications (SCPs) and 112 multiple-country publications (MCPs). The United States followed with 397 articles (19.1%), including 334 SCPs and 63 MCPs. Subsequent contributors included Japan, Australia, the United Kingdom, Canada, South Korea, Spain, Brazil, and Sweden. Notably, Belgium exhibited the highest proportion of international collaboration, with MCPs accounting for 76% of its output (19 MCPs versus 6 SCPs) (**Figure 3C**). The United States demonstrated the greatest research impact, accruing 22,299 total citations and achieving the highest citations per publication (CPP) rate of 56.16. Despite China’s dominance in publication volume, its citation metrics revealed a substantial gap relative to the United States (**Figure 3B**). Co-authorship network analysis identified the United Kingdom (total link strength [TLS] = 413), the United States (TLS = 364), and Australia (TLS = 241) as the most collaborative nations within the field (**Figure 3D**).

### 3.3 Analysis of institutions

**Figure 4:**
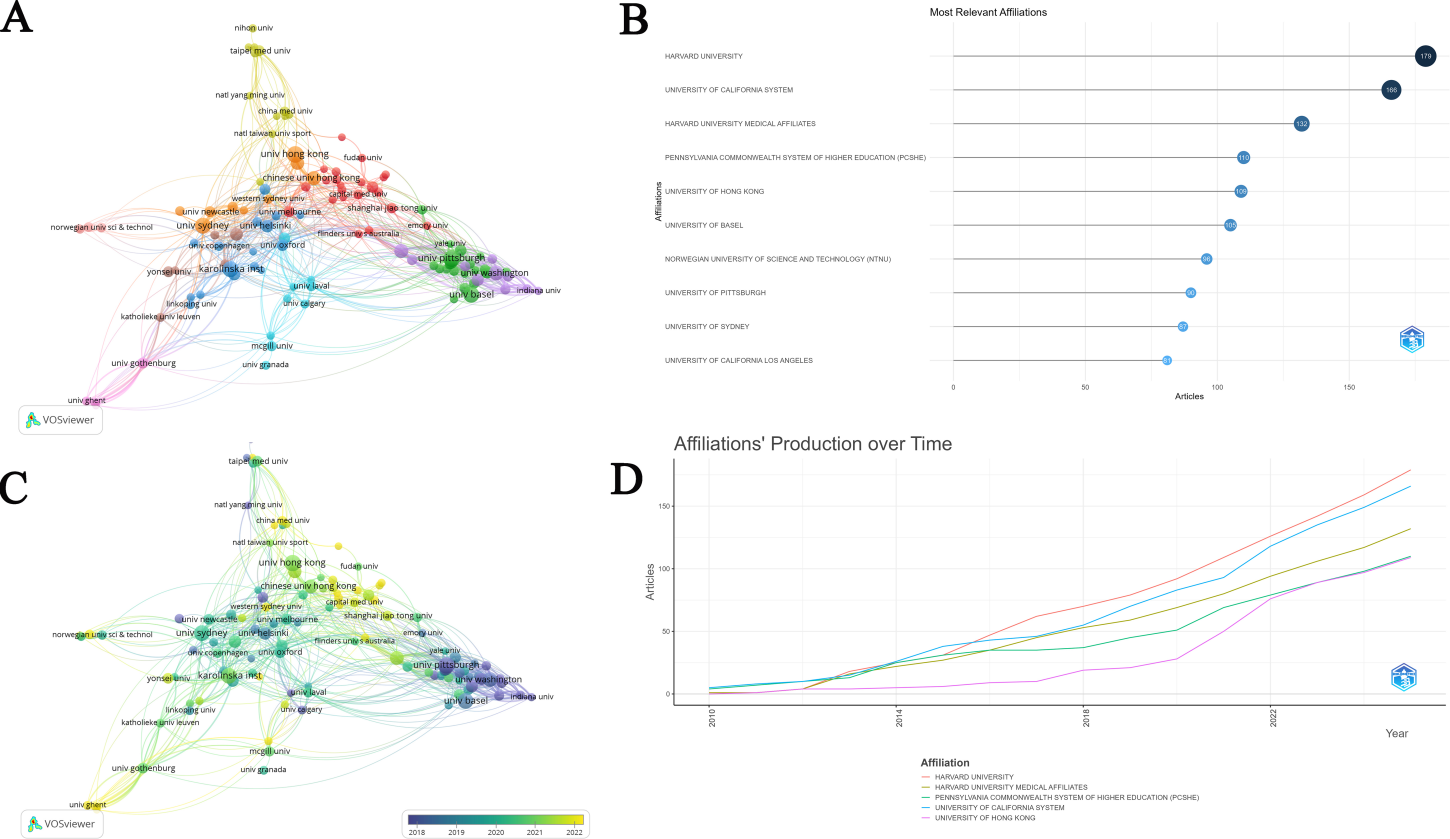
Institutional networks and productivity. **(A)** Network visualization of institutional collaborations. **(B)** The top 10 most contributing institutions. **(C)** The overlay visualization of bibliographic coupling between institutions. **(D)** Annual publication trends of the top five institutions.

A total of 2,147 institutions contributed to the physical activity and insomnia literature. Of these, 116 institutions produced more than 10 publications and were selected for co-authorship network visualization and bibliographic coupling analysis (**Figures 4A, C**). The top 10 most productive institutions were led by Harvard University (n = 179), followed by the University of California System (n = 166) and the Harvard University Hospital (n = 132) (**Figure 4B**). Bibliographic coupling analysis of institutions with ≥10 publications revealed distinct temporal patterns: the University of Washington, University of Pittsburgh, and University of Basel demonstrated higher publication output during earlier periods of the field’s development, whereas Ghent University, Beijing Sport University, and China Medical University have shown increased productivity in recent years (**Figure 4C**). Analysis of the top five institutions revealed a marked surge in Harvard-affiliated publications after 2018, with this institution system maintaining a predominant share (exceeding 50%) of output among the leading cohort; concurrently, other North American and European institutions exhibited gradual growth trajectories. Post-2023 data indicated a plateau in combined output from these elite institutions, suggesting an entrenched pattern of North American dominance within the upper tier of the field (**Figure 4D**). Given that publication count alone provides an incomplete measure of institutional impact, supplementary analyses examined citation metrics and total link strength (TLS) (**Table 1**). Comprehensive assessment of these indicators indicates that the University of Washington and University of Pittsburgh have made particularly substantial contributions to advancing this research domain.

**Table 1:** Bibliometric profile of leading contributing institutions: publications, citations, and total link strength.

No.	Country	Documents	Country	Citations	Country	TLS
1	Harvard University	179	UCL	2955	University of Washington	93
2	University System of California	166	King’s College London	2554	Harvard Medical School	75
3	Harvard University Hospital	132	South London & Maudsley NHS Foundation Trus	2156	Karolinska Institute	66
4	Pennsylvania Higher Education System (PCSHE)	110	University of Pittsburgh	2095	University of Minnesota	63
5	The University of Hong Kong	109	Massachusetts General Hospital	1779	Fred Hutchinson Cancer Research Center	62
6	University of Basel	105	Karolinska Institute	1762	University of Pennsylvania	61
7	Norwegian University of Science and Technology (NTNU)	96	Northwestern University	1717	University of Pittsburgh	59
8	University of Pittsburgh	90	University of Basel	1620	University of Hong Kong	58
9	University of Sydney	87	University of Washington	1620	University of California	57
10	Karolinska Institute	81	University of Sydney	1586	Brigham and Women’s Hospital	57

TLS, total link strength.

### 3.4 Analysis of journals

**Figure 5:**
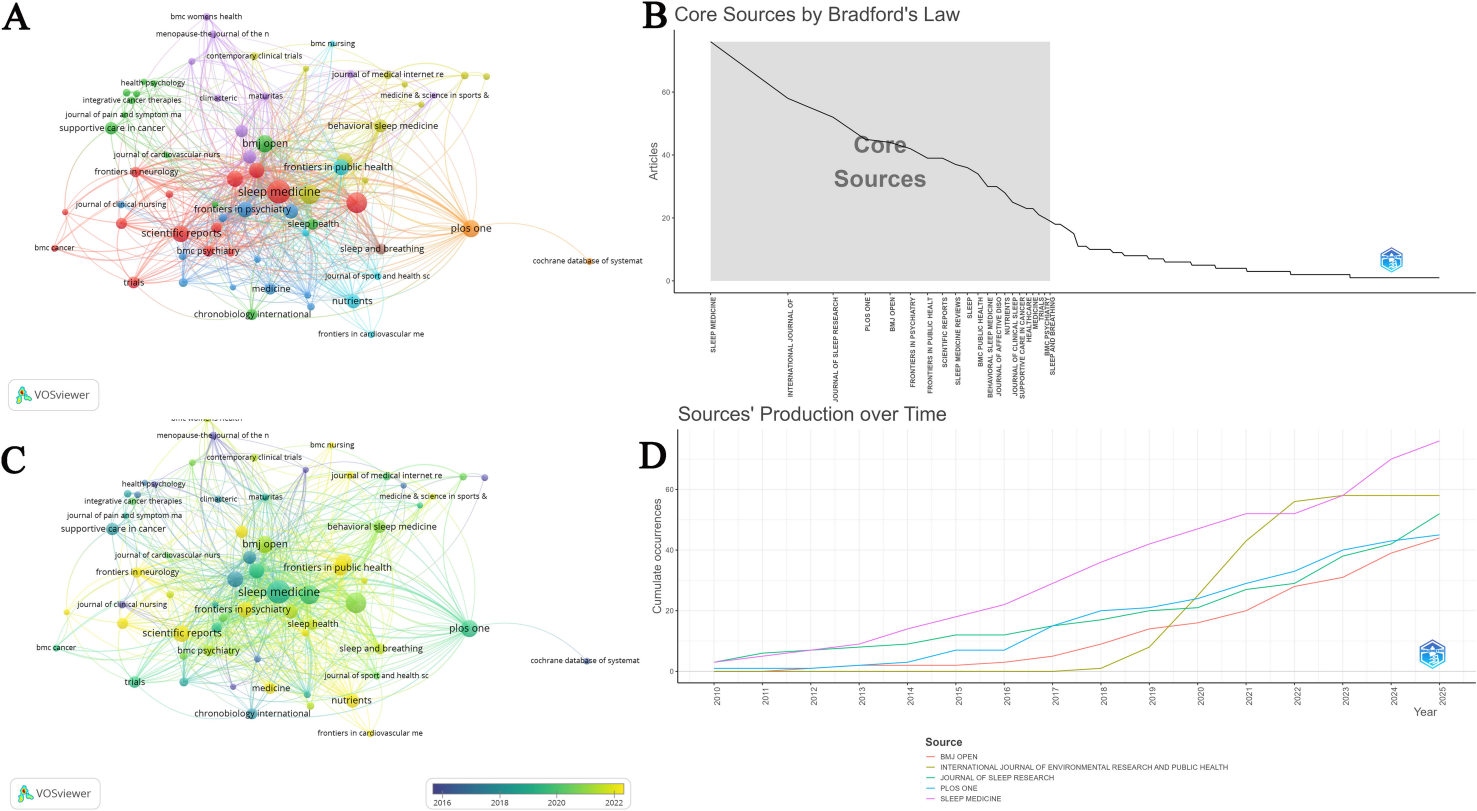
Analysis of journals. **(A)** Network visualization illustrating academic journals collaborations. **(B)** Core journals by Bradford’s law. **(C)** The overlay visualization of bibliographic coupling between academic journals. **(D)** Annual publication trends of the top five journals.

As shown in **Table 2**, *Sleep Medicine* demonstrated the highest academic impact in this domain, exhibiting the highest *h*-index (30), which strongly correlated with its leading position in both publication volume (*n* = 76) and total citations (*n* = 2,928). Ranked second is *Sleep Medicine Reviews*, with an *h*-index of 30, contributing 37 articles, and accumulating 3,938 citations. This pattern persisted across the journal landscape, indicating a consistent positive correlation between journal *h*-indices and both publication output and citation frequency. A total of 668 journals published articles on physical activity and insomnia. Of these, 90 journals contributed more than five articles each. Co-authorship and bibliographic coupling analyses were conducted on these 90 journals to generate network visualizations (**Figures 5A, C**). Bibliographic coupling analysis revealed that journals with higher publication counts during earlier periods included *Menopause* (The Journal of The Menopause Society), *Journal of the American Geriatrics Society*, and *JMIR mHealth and uHealth*. By contrast, *Frontiers in Public Health*, *Scientific Reports*, and *BMC Public Health* have exhibited higher publication volumes in recent years (**Figure 5C**). **Figure 5B** illustrates that the Bradford core comprises 21 high-quality journals, representing 3.35% of the total corpus and collectively accounting for 745 articles. Prominent journals within this nucleus include *Sleep Medicine*, *Sleep Medicine Reviews*, *Sleep*, *Journal of Sleep Research*, and *PLOS ONE*, all characterized by high *h*-indices. The top five journals exhibited varying growth trajectories after 2015, with *International Journal of Environmental Research and Public Health* demonstrating particularly pronounced growth in recent years (**Figure 5D**), potentially reflecting substantially increased research productivity in this field.

**Table 2:** Top 10 most influential journals.

Rank	Journal	h_index	TC	NP
1	Sleep Medicine	30	2928	76
2	Sleep Medicine Reviews	30	3938	37
3	Sleep	26	1885	36
4	Journal of Sleep Research	22	4270	52
5	PLOS ONE	22	2545	45
6	International Journal of Environmental Research and Public Health	21	1510	58
7	Journal of Affective Disorders	18	1490	30
8	Journal of Clinical Sleep Medicine	16	1059	25
9	Scientific Reports	15	632	39
10	Supportive Care in Cancer	15	686	24

TC, total citations; NP, number of publications.

### 3.5 Author keywords frequency and trend topics

At the author level, our analysis revealed the relationship between publication metrics and scholarly impact (**Table 3**). Specifically, the *h*-index and total citation count served as primary indicators of academic influence, whereas publication volume reflected research productivity. BRAND S and GERBER M achieved the highest *h*-indices (*h* = 18), with total citations of 1,527 and 1,491, respectively, and comparable publication outputs of 30 and 29 articles.

**Table 3:** Top 10 most influential authors.

Rank	Author	h_index	TC	NP
1	Brand S	18	1527	30
2	Gerber M	18	1491	29
3	Pühse U	15	1285	25
4	Holsboer-Trachsler E	14	1274	18
5	Duncan MJ	11	669	16
6	Buysse DJ	11	735	14
7	Lacroix AZ	10	532	10
8	Lindberg E	10	331	14
9	De Mello MT	9	482	9
10	Ensrud KE	9	500	9

TC, total citations; NP, number of publications.

**Figure 6:**
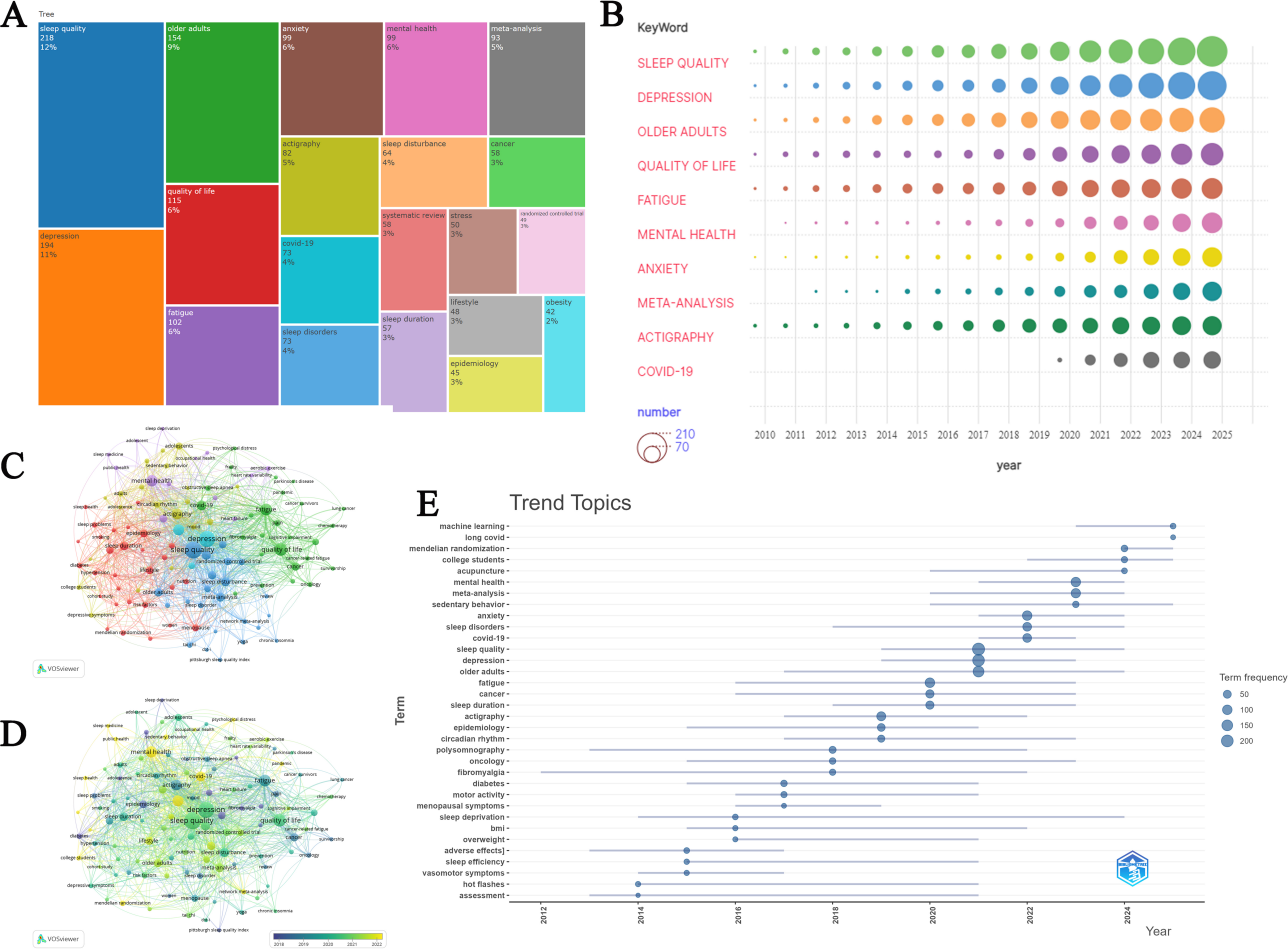
Analysis of keywords. **(A)** Tree map of the most frequent author keywords. **(B)** Annual publication trends of the top 10 keywords. **(C)** Network visualization illustrating of keywords. **(D)** The overlay visualization of keywords. **(E)** Trend topics in the research domain of PA and Insomnia.

Analysis of the top 20 author keywords in the physical activity and insomnia domain is presented in **Figure 6A**. The three most frequent author keywords were “sleep quality” (*n* = 218), “depression” (*n* = 194), and “older adults” (*n* = 154), accounting for 12%, 11%, and 9% of all keyword occurrences, respectively. **Figure 6B** displays a bubble chart illustrating the temporal evolution of high-frequency keywords from 2010 to 2025. Overall, COVID-19 emerged as a prominent research focus in recent years. The dataset comprised 1,528 distinct keywords, of which 122 occurred with a frequency exceeding 10. Network visualization and bibliographic coupling analyses of these 122 keywords are presented in **Figures 6C, D**.

Thematic evolution analysis revealed distinct temporal patterns in research foci (**Figure 6E**). From 2015 to 2024, research topics exhibited marked dynamism. Early established themes such as “polysomnography” and “fatigue” gained substantial traction during 2012–2016, with polysomnography—as an objective sleep assessment methodology—maintaining prolonged relevance through 2023. During 2019–2021, three core directions coalesced: “sleep quality,” “depression,” and “older adults,” intensifying rapidly following the COVID-19 outbreak. The most recent hotspot phase (2021–2023) shifted toward “long COVID,” “mental health,” “Mendelian randomization,” “college students,” and “machine learning,” marking a paradigm shift from acute pandemic stress to long-term sequelae and genetic causality. Notably, “sleep quality” persisted as a dominant theme throughout the entire observation period (*n* = 206), maintaining sustained academic attention. This thematic evolution demonstrates the field’s immediate responsiveness to public health emergencies while reflecting the converging imperatives of global population aging and precision medicine, driving urgent demands for brain health and quality-of-life improvements.

**Figure 7:**
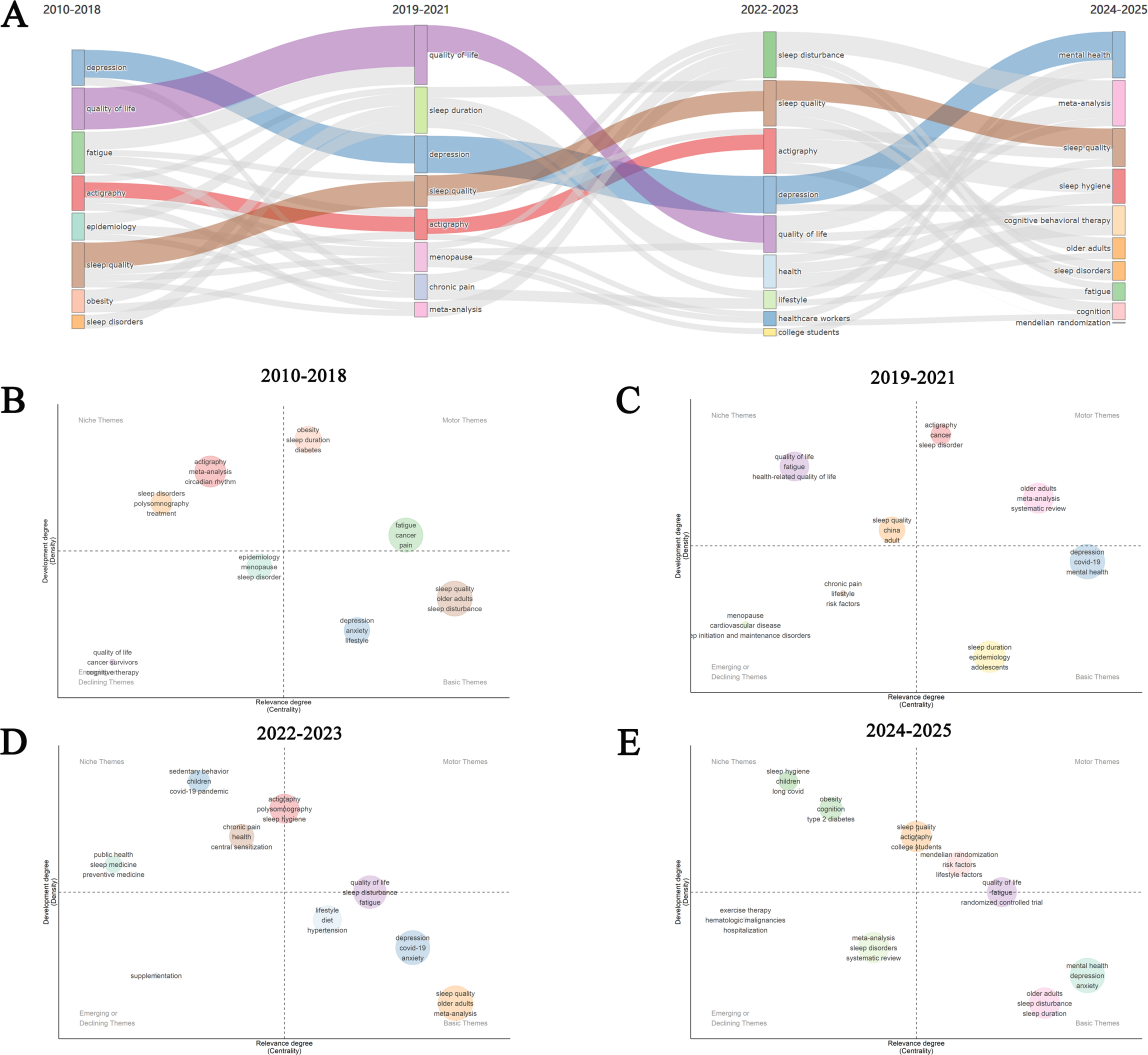
The evolution trend of keywords. **(A)** The changes and internal connections of keywords in different time periods. Thematic evolution within PA and Insomnia in the periods 2010–2018 **(B)**, 2019–2021 **(C)**, 2022–2023 **(D)**, and 2024–2025 **(E)**.

**Figure 7A** illustrates the evolution and interrelationships of research themes across distinct temporal segments from 2010 to 2025. Nodes represent keywords, with size proportional to their frequency of occurrence; edges denote co-occurrence relationships between keywords in the literature, with thickness indicating the strength of these associations. To ensure balanced representation across phases, the study period was stratified into four intervals (2010–2018: n = 657; 2019–2021: n = 584; 2022–2023: n = 471; 2024–2025: n = 512), a segmentation consistent with automated temporal partitioning by Biblioshiny software. Keyword networks within each interval demonstrate the shifting research priorities characteristic of that period.

The early period (2010–2018) exhibited a polycentric configuration, with five primary thematic origins—actigraphy, fatigue, depression, sleep quality, and epidemiology—radiating outward toward circadian rhythms, cancer-related fatigue, psychiatric comorbidities, subjective sleep quality, and population-based cross-sectional surveys, without coalescing into a distinct central cluster. Between 2019 and 2021, the field underwent significant thematic convergence, marked by the crystallization of a tripartite structure centered on the sleep quality–depression–older adults axis, accompanied by four parallel branches: quality of life, actigraphy, menopause, and chronic pain. The COVID-19 pandemic catalyzed the rapid ascendance of mental health and sleep disorders, shifting the research paradigm from conventional survey methodologies toward objective wearable monitoring (actigraphy) and evidence synthesis (meta-analysis). During 2022–2023, the thematic network underwent further differentiation, forming high-density chain pathways (e.g., depression→mental health; sleep quality→meta-analysis; chronic pain→lifestyle). Notably, Mendelian randomization emerged as a novel insertion point via the sleep quality node, signaling the emergence of causal inference and genetic dimensions as new vectors of methodological expansion. In the 2024–2025 interval, the research landscape demonstrated increased consolidation, with long COVID, college students, and mobile phone-based digital health emerging as novel terminal nodes. Mendelian randomization and sleep hygiene functioned as high-stability bridging terms, re-anchoring the three traditional cores—sleep quality, depression, and quality of life—to a precision public health framework. This evolutionary trajectory demonstrates that sleep-emotion research has progressed from early descriptive epidemiology, through pandemic-responsive phases, toward an era of multidisciplinary integration characterized by “genetic-behavioral-digital” interventions. This progression maintains a sustained focus on neuro-psychiatric-metabolic interaction mechanisms, addressing the urgent imperative for brain health and quality-of-life enhancement amid global population aging and escalating chronic disease burdens.

**Figures 7B–E** illustrate the evolutionary trajectories of research themes within the physical activity and insomnia domain across distinct temporal intervals, with thematic maps constructed via keyword clustering providing a strategic overview of the field’s intellectual structure. Each temporal map is partitioned into four quadrants defined by two axes: the horizontal axis denotes centrality (the degree of a theme’s relevance to the broader field), while the vertical axis represents density (the degree of a theme’s internal development). Centrality is quantified using betweenness centrality within the co-occurrence network. This metric measures the extent to which a node functions as a structural “bridge” connecting other nodes; higher values indicate that a theme plays a more critical intermediary role between disparate research areas and exhibits stronger relevance to the field’s core knowledge base. Density is calculated based on the theme’s internal cohesion, specifically defined as the ratio of the sum of edge weights for internal co-occurrences to the theoretical maximum possible weight sum. Higher density values signify stronger internal linkages among keywords within the theme, reflecting a higher level of development and maturity for that specific research area (18, 19).

In **Figure 7B** (2010–2018), the cluster Obesity–sleep duration–diabetes constituted a metabolically driven motor theme, representing the domain’s dynamic core; methodologically and disease-specific clusters (actigraphy, meta-analysis, sleep disorders) were positioned as specialized themes (high density, low centrality), indicating well-developed yet peripheral research streams; behavioral interventions and quality-of-life concepts occupied the emerging/redeveloping quadrant (low centrality, low density), signifying nascent conceptual formations; while cross-domain indicators (depression, sleep quality, older adults) functioned as basic/transversal themes (high centrality, low density), serving as foundational bridges across subfields. This configuration marked the field’s rapid transition toward metabolically-oriented, precision-based, and digitally-enabled interventions. **Figure 7C** (2019–2021) reveals that actigraphy, cancer, sleep disorders, and older adults converged in the high-centrality, high-density quadrant, constituting motor themes and demonstrating the bifurcated core of methodological innovation and population-specific research. Simultaneously, quality of life, fatigue, and sleep quality clustered as specialized themes, suggesting the maturation of fine-grained outcome assessment. Conversely, chronic pain, lifestyle, risk factors, menopause, and cardiovascular disease populated the emerging/redeveloping quadrant, indicating multi-system intervention concepts awaiting integration; whereas depression, COVID-19, mental health, and sleep duration distributed across basic themes, maintaining transversal associations across populations and disease entities. This distribution signaled the field’s rapid transition from pandemic-responsive research to precision-oriented, evidence-based inquiry. During 2022–2023 (**Figure 7D**), actigraphy, polysomnography, sleep hygiene, quality of life, sleep disturbance, and fatigue co-occupied the motor theme quadrant, indicating the consolidation of an objective monitoring–behavioral intervention–outcome assessment continuum as the domain’s central engine. Sedentary behavior, children, COVID-19 pandemic, chronic pain, health, central sensitization, public health, sleep medicine, and preventive medicine clustered as specialized themes, reflecting the post-pandemic intensification of multi-system, life-course prevention research. Notably, supplementation alone occupied the emerging/redeveloping quadrant, suggesting that nutritional interventions remained in preliminary validation phases. Meanwhile, lifestyle, diet, hypertension, depression, COVID-19, anxiety, sleep quality, older adults, and meta-analysis distributed across basic themes, continuing to provide transversal associations and methodological scaffolding across populations and conditions. This configuration marked the field’s acceleration toward precision prevention and multidimensional intervention. In 2024–2025 (**Figure 7E**), Mendelian randomization, risk factors, lifestyle factors, sleep quality, actigraphy, college students, quality of life, fatigue, and randomized controlled trial constituted the motor themes, demonstrating the fusion of genetic causal inference with behavioral-digital interventions as the primary driving force. Sleep hygiene, children, long COVID, obesity, cognition, and type 2 diabetes clustered as specialized themes, indicating sustained specialization in post-pandemic metabolic-cognitive and pediatric-adolescent research tracks. Conversely, meta-analysis, sleep disorders, systematic review, exercise therapy, hematologic malignancies, and hospitalization populated the emerging/redeveloping quadrant, reflecting paradigm updates in evidence synthesis methodologies and the nascent application of exercise interventions in hematologic malignancies and inpatient settings. Mental health, depression, anxiety, older adults, sleep disturbance, and sleep duration remained distributed across basic themes, preserving their transversal relevance across populations and disease categories. Collectively, these strategic diagrams reveal that sleep and mental health research has transitioned from associational description to an integrated “genetic causality-precision behavior-multidimensional outcomes” paradigm by 2024–2025. Motor themes now provide dual theoretical-methodological engines; specialized themes demonstrate intensified focus on subpopulations; basic themes maintain cross-domain infrastructural support; and emerging themes point toward novel directions in evidence synthesis and clinical translation.

### 3.6 Cross-database validation with scopus

To assess the robustness of our primary findings and mitigate potential biases inherent to a single database, we conducted cross-validation using an independent dataset retrieved from Scopus. Although the initial search yield differed substantially between Scopus (4,754 records after screening) and WoS (2,224 records after screening), reflecting their fundamental scope divergences—WoS emphasizes high-impact journals in natural sciences, engineering, and social sciences with more stringent selection criteria, whereas Scopus adopts a more inclusive coverage spanning 27 disciplines including life sciences, social sciences, natural sciences, and medicine with broader inclusion of humanities and emerging fields—comparative analysis of key bibliometric indicators revealed a high degree of concordance.

A comparative examination of annual publication trends across the two databases (**Supplementary Figure 2**) demonstrated markedly similar growth trajectories. Both datasets exhibited a pronounced upward trend in publication volume over the past decade. This strong consistency in temporal patterns substantiates that the observed publication growth in the PA-insomnia field represents a genuine and reproducible phenomenon, rather than an artifact of database-specific coverage.

A comparative top-20 keyword analysis indicated substantial thematic overlap between Scopus and WoS (approximately 85%–90% concordance), with both databases converging on core themes including depression, anxiety, sleep quality, older adults, and mental health (**Supplementary Figure 3**). Nevertheless, notable disciplinary orientation differences emerged: Scopus exhibited a stronger clinical medicine emphasis, as evidenced by higher proportions of clinically oriented terms such as depression (12%), obesity (5%), and COVID-19 (6%); conversely, Web of Science demonstrated greater emphasis on methodological and public health perspectives, with sleep quality ranking first (12%) and relatively prominent representation of methodologically oriented terms including actigraphy (5%), randomized controlled trial (3%), and epidemiology (3%). While research hotspots were broadly convergent across databases, divergent characteristics were evident in disciplinary distribution and terminological preferences.

Longitudinal thematic evolution analysis further corroborated this high concordance (approximately 85%–90% similarity) between databases (**Supplementary Figure 4**). Both Scopus and Web of Science exhibited a highly synchronized four-stage paradigmatic transition: (i) clinical disease-oriented phase (2010–2018); (ii) sleep-function outcome integration phase (2019–2021); (iii) thematic differentiation and methodological infiltration phase (2022–2024); and (iv) evidence-based integration and psychosocial comprehensive model phase (2024–2025). Depression and quality of life constituted stable cross-stage thematic backbones in both databases; sleep quality ascended to a central node during the intermediate phase and persisted through the terminal period; and actigraphy was concurrently incorporated as an objective assessment tool within the methodological framework. During the later phase, both databases concurrently evidenced emergent directions including lifestyle, Mendelian randomization, and sleep disorder subtypes, reflecting synchronized field-level attention toward modifiable behavioral factors, genetic epidemiological approaches, and sleep phenotype refinement. Database-specific divergences were also apparent: Scopus incorporated neurodegenerative and neuroendocrine nodes such as Alzheimer’s disease and cortisol, indicating a broader clinical disease spectrum; whereas Web of Science emphasized psychological intervention and population-specific orientations including cognitive behavioral therapy, college students, and healthcare workers, manifesting more pronounced public health and applied psychology characteristics. Collectively, both databases convergently reveal an overarching field-level evolution toward a multidimensional integrative model encompassing sleep, psychological, behavioral, and methodological dimensions.

## 4 Discussion

The physical activity and insomnia research domain has emerged as a prominent scientific focus. Between 2010 and 2025, the literature experienced substantial growth, evidenced by significant increases in both annual publication output and cumulative citations. Our bibliometric analysis characterized the field’s intellectual structure across multiple dimensions, encompassing citation patterns, journal distributions, institutional productivity, geographic contributions, reference co-citation networks, author keyword frequencies, and thematic evolution trajectories. Furthermore, we identified prevailing research trends and delineated the domain’s thematic progression throughout the 15-year period.

Trend topic analysis captures the iterative evolution of research priorities in the physical activity and insomnia domain. Influential themes began to emerge prominently from 2012 onward, with temporal evolution delineating four distinct phases (2010–2018, 2019–2021, 2022–2023, and 2024–2025). During the initial phase (2010–2018), research priorities centered on the establishment and validation of measurement instruments. The co-occurrence of keywords such as polysomnography, actigraphy, and sleep efficiency indicates that the primary objective involved calibrating sleep assessment methodologies. Concurrently, terms including menopausal symptoms, fibromyalgia, and oncology suggest that study populations were predominantly restricted to menopausal women and chronic disease cohorts, with research aims focused on establishing concordance between the Pittsburgh Sleep Quality Index (PSQI) and objective monitoring devices rather than elucidating causal mechanisms (20). The early emergence of Alzheimer’s disease and sleep deprivation within this period indicates the incorporation of elderly cohorts into baseline observational frameworks, thereby establishing foundational datasets for subsequent longitudinal investigations (21). In the second phase (2019–2021), the COVID-19 global pandemic catalyzed a rapid thematic shift toward the “sleep-emotion-stress” nexus. The high-frequency emergence of terms such as circadian rhythm, epidemiology, fatigue, and older adults signified an expansion of research contexts from laboratory settings to community and clinical environments, with particular attention to the bidirectional interactions between circadian disruption and pandemic-related psychological stress (22). The concurrent ascendance of quality of life and depression indicated the incorporation of subjective health outcomes as core endpoints, while the co-occurrence of actigraphy and sleep duration reflected the mainstream adoption of wearable devices as primary data collection instruments, providing technical infrastructure for large-scale cross-sectional investigations (23). During the third phase (2022–2023), systematic reviews and intervention meta-analyses emerged as the dominant methodological paradigm. The clustering of keywords including meta-analysis, systematic review, anxiety, sleep disorders, and mental health demonstrated the field’s transition toward secondary synthesis of the substantial data accumulated during the pandemic period, specifically validating bidirectional causal models between anxiety and insomnia (24). Notably, registration numbers for digital cognitive behavioral therapy for insomnia (dCBT-I) and remote exercise interventions surged during this interval, signaling a strategic pivot from descriptive association studies toward evidence integration and the development of scalable digital therapeutics (25). In the fourth phase (2024–2025), genetic causality and youth cognition emerged as novel growth vectors. The inaugural appearance of Mendelian randomization alongside college students, cognitive impairment, and long COVID indicated a methodological shift toward utilizing genetic instrumental variables to disentangle the causal directionality between physical activity, sleep, and cognitive function—thereby addressing the causal inference limitations inherent to prior cross-sectional evidence (26). Concurrently, subjective scales (sleep quality, depression) were retained as phenotypic anchoring tools, establishing a multi-dimensional evidentiary chain comprising genetic instruments-questionnaire-wearable modalities. This configuration portends the field’s progression toward an era characterized by precision medicine and digitally-enabled interventions (23).

Comparative analysis of keyword temporal trajectories and strategic quadrant positioning reveals remarkable alignment across phase delineation, thematic categorization, and evolutionary logic. During 2010–2018, peak keyword frequencies coalesced around metabolic constructs (obesity, sleep duration, diabetes), aligning precisely with the motor theme quadrant in **Figure 7B**. This configuration underscores the early-phase emphasis on metabolic indicators and psychometric validation of assessment tools. The 2019–2021 interval witnessed significant ascendance of terms including actigraphy, older adults, COVID-19, and mental health, congruent with the “methodology-population bifurcated core” depicted in **Figure 7C**. This reflects the field’s rapid pivot toward evidence synthesis and the characterization of sleep-emotion comorbidities amid pandemic-related psychological stress. During 2022–2023, the co-occurrence of sleep hygiene, quality of life, and fatigue alongside the actigraphy–polysomnography continuum corresponded to the “objective monitoring–behavioral intervention–outcome evaluation” motor theme in **Figure 7D**, signifying the crystallization of a closed-loop intervention paradigm. In 2024–2025, the inaugural clustering of Mendelian randomization, college students, and randomized controlled trial mapped onto the “genetic causality–behavioral–digital intervention” motor core in **Figure 7E**, demarcating the field’s formal transition into an integrated era of causal inference and precision digital therapeutics. Notably, both basic themes (epidemiology, sleep quality, older adults) and specialized themes (children, long COVID, obesity) demonstrated temporal stability across keyword evolution and quadrant distributions, substantiating their intrinsic coherence within the intellectual structure. Collectively, keyword trajectories and strategic diagrams exhibit mutual corroboration, jointly delineating a four-stage evolutionary arc: metabolic measurement → pandemic stress responsiveness → preventive intervention → genetic precision. This convergent evidence provides robust bibliometric support for the domain’s methodological advancement and theoretical paradigm shift.

Thematic evolution analysis enables the identification of emerging research frontiers within the physical activity and insomnia domain. Focusing specifically on the 2024–2025 interval, themes including Mendelian randomization, risk factors, lifestyle factors, sleep quality, actigraphy, college students, quality of life, fatigue, and randomized controlled trial occupied the upper-right Motor Themes quadrant, exhibiting elevated levels of both internal cohesion (density) and external centrality. This positioning indicates that these research streams have not only established mature endogenous knowledge structures but also maintain extensive cross-linkages with external research areas, functioning as the domain’s current intellectual core with substantial capacity for knowledge diffusion and future growth potential. Mendelian randomization (MR) investigations have established independent and significant causal relationships between modifiable upstream lifestyle factors—including dietary patterns, physical activity levels, sleep duration, smoking and alcohol consumption, and sedentary behavior—and downstream risk factors such as obesity, metabolic syndrome, cognitive decline, daytime sleepiness, depression, and chronic pain. These causal inferences remain robust against confounding by immutable characteristics (e.g., genetic background, socioeconomic status, or inflammatory predisposition) and reverse causality. For instance, Chen et al. (27) employed MR designs to demonstrate that higher physical activity levels are significantly associated with longer leukocyte telomere length, suggesting protective effects against aging-related diseases (27). Similarly, Jiang et al. (28) utilized two-sample MR to reveal that regular physical activity significantly reduces the risk of poor functional outcomes post-stroke (28). Furthermore, Guan et al. (29) reported in their systematic review and MR meta-analysis that smoking, alcohol consumption, and sedentary behavior demonstrate positive causal associations with low back pain risk (29). Collectively, these findings indicate that lifestyle factors function as upstream modifiable exposures that amplify or suppress core downstream risk factors—spanning obesity, metabolic dysregulation, psychiatric disorders, and frailty—through genetically supported causal pathways, thereby determining the trajectory of chronic disease onset and progression. Research centered on the sleep quality–actigraphy–college students nexus typically engages undergraduate populations aged 18–25 years across diverse academic disciplines (medicine, engineering, arts). Methodologically, these studies employ wrist-worn accelerometers for continuous monitoring (≥7 days), generating objective metrics including sleep efficiency, sleep onset latency, total sleep time, nocturnal awakening frequency and duration, and circadian phase markers. These parameters are analyzed alongside subjective Pittsburgh Sleep Quality Index (PSQI) scores to construct comprehensive phenotypic profiles of sleep quality. Empirical findings consistently demonstrate that mobile phone/social media addiction, sedentary behavior, and pandemic-related online learning stress significantly impair sleep efficiency, delay sleep onset, and increase nocturnal awakenings. Conversely, moderate-intensity aerobic exercise, yoga, or walking interventions (1–8 weeks duration) yield measurable improvements in actigraphic sleep efficiency and reduced sleep latency, concordant with subjective PSQI amelioration (30, 31). Extended investigations integrating actigraphy with mental health scales, cognitive assessments, or electroencephalography further elucidate the longitudinal impact of sleep quality on depression, anxiety, and executive function (10). Collectively, this literature establishes actigraphy as the objective core methodology, intersecting with subjective scales and behavioral-psychological variables to systematically characterize the multidimensional determinants of sleep quality and the efficacy of exercise interventions in collegiate populations. Integrating intervention studies indexed by keywords quality of life, fatigue, and randomized controlled trial, convergent evidence demonstrates that exercise, mind-body, and multimodal interventions significantly ameliorate cancer-related fatigue (CRF) while concomitantly enhancing quality of life. For example, Schad et al. (32) conducted a randomized controlled trial demonstrating that a 12-week Argentine tango program significantly reduced CRF and improved quality of life among breast cancer survivors (32). Similarly, Lin et al. (33) investigated yoga interventions in cancer survivors, identifying fatigue reduction as a significant mediator in the pathway linking yoga to improved quality of life (33). Consequently, exercise and mind-body interventions targeting fatigue represent high-quality, evidence-based strategies for enhancing quality of life in oncology populations.

In the upper-left quadrant (Niche Themes), keywords such as sleep hygiene, children, long COVID, obesity, cognition, and type 2 diabetes exhibit high external centrality yet low internal density. This configuration indicates that while these themes possess relatively weak internal cohesion, they maintain robust connections with other research streams within the domain. Current evidence indicates that interventions for sleep disturbances in long COVID require sleep hygiene as the core component of multi-modal protocols. A systematic review noted that non-pharmacological insomnia interventions for children typically bundle sleep hygiene education with regular sleep-wake scheduling, screen time restriction, and relaxation training. Although the isolated effect of sleep hygiene remains difficult to disentangle from bundled interventions, it is regarded as a safe and feasible foundational module (34). In adult long COVID cohorts, randomized controlled trials further demonstrate that group-based cognitive behavioral therapy for insomnia (CBT-I) emphasizing sleep hygiene principles significantly improves sleep onset latency, sleep efficiency, and daytime functioning within three months, with effects sustained at six-month follow-up (35). Although no randomized controlled trials specifically targeting pediatric long COVID have isolated the efficacy of sleep hygiene, the convergence of general pediatric evidence and adult long COVID data suggests that family-based sleep hygiene guidance—including fixed bedtimes and wake times, cessation of electronic screen use one hour before sleep, and maintenance of a quiet, dark bedroom environment—should constitute the first-line intervention for sleep disturbances in children with long COVID, providing a safe baseline for subsequent cognitive behavioral or pharmacological treatments. Evidence further indicates a bidirectional, mutually reinforcing negative cycle among obesity, cognitive decline, and type 2 diabetes. Obesity exacerbates insulin resistance, promotes chronic low-grade inflammation, and induces circadian disruption, thereby directly impairing neuronal plasticity and executive function (36). Conversely, type 2 diabetes amplifies glycemic fluctuations and microvascular lesions, significantly elevating the risk of cognitive deterioration (37). In the reverse direction, obesity-related lifestyle factors—including short sleep duration, poor sleep quality, and sedentary behavior—not only accelerate adiposity but also activate the hypothalamic-pituitary-adrenal (HPA) axis, thereby intensifying cravings for high-sugar, high-fat foods and fostering a bidirectional cognitive-metabolic deterioration (38, 39). Consequently, comprehensive lifestyle modifications integrating glycemic management with sleep and exercise interventions for obese populations are regarded as critical strategies for delaying cognitive decline and reducing type 2 diabetes incidence.

In the lower-right quadrant (Basic Themes), keywords including mental health, depression, anxiety, older adults, sleep disturbance, and sleep duration exhibited high internal density yet low external centrality. This configuration indicates that these themes maintain strong internal coherence while operating with relative independence from other research streams, signifying their status as autonomous yet significant research directions during 2024–2025. Mental health, depression, and anxiety frequently demonstrate bidirectional associations with sleep disturbances, whereas physical activity has been consistently identified as a modifiable factor capable of disrupting this vicious cycle. For instance, Kline et al. (40) demonstrated that sustained high levels of physical activity significantly improve sleep continuity and depth among middle-aged and older women, thereby buffering against depression and anxiety risks (40). Zhai et al. (30) further elucidated that physical activity indirectly enhances sleep quality through reduced perceived stress, subsequently alleviating anxiety and depressive symptoms in university students (30). Additionally, Su et al. (41) confirmed that anxiety and depression exert chain mediation effects between physical activity and sleep quality, suggesting that improved activity levels can simultaneously attenuate both psychopathological pathways (41). Collectively, these findings indicate that maintaining regular physical activity serves as an effective non-pharmacological strategy for promoting overall psychological health, functioning through both direct sleep optimization and indirect mood regulation mechanisms. Regarding older adults, bidirectional associations similarly exist between sleep disturbance and sleep duration, wherein both short sleep (≤6 hours) and long sleep (≥8 hours) correlate with elevated sleep disorder prevalence, with this relationship being particularly pronounced among older women. Liu and Yang (42), utilizing cross-sectional data from Chinese community populations, identified that short sleepers exhibited significantly elevated risks of sleep-onset difficulties, sleep maintenance problems, and daytime functional impairment, whereas long sleepers more frequently reported early morning awakenings and sleep fragmentation (42). Furthermore, Skarpsno et al. (43), leveraging longitudinal data from the Norwegian HUNT study, demonstrated that baseline sleep durations of <6 hours or >9 hours predicted exacerbated insomnia symptoms after 10 years in elderly cohorts, with this relationship remaining independent of baseline physical activity levels (43). These convergent findings suggest that maintaining a sleep duration of 7–8 hours may represent an important non-pharmacological target for reducing sleep disorder risk among older adults.

In the lower-left quadrant (Emerging or Declining Themes), keywords including meta-analysis, sleep disorders, systematic review, exercise therapy, hematologic malignancies, and hospitalization demonstrate low internal density and low external centrality. This configuration suggests that while these themes may have constituted significant research foci historically, they have experienced declining attention during 2024–2025, gradually transitioning toward marginalization within the current research landscape. Synthesizing recent evidence from meta-analyses and systematic reviews, the efficacy of sleep disorder interventions has been repeatedly validated across diverse populations and therapeutic modalities. Atoui et al. (9) conducted a systematic review and meta-analysis examining the daily association between sleep and physical activity, demonstrating that increased daily physical activity significantly improves subjective sleep quality (9). Furthermore, Banno et al. (44), in a study published in PeerJ, affirmed that exercise improves sleep quality; their meta-analysis of multiple randomized controlled trials confirmed that regular exercise confers a moderate overall therapeutic effect on various sleep disorders (44). Concurrently, digital health modalities have gained empirical support. An umbrella review published in NPJ Digital Medicine (Singh et al., 45) demonstrated that electronic and mobile health interventions consistently enhance sleep-related behaviors across diverse age groups and health statuses, with efficacy comparable to traditional face-to-face approaches (45). Collectively, these systematic reviews and meta-analyses indicate that both physical activity promotion and digital behavioral interventions constitute safe and effective non-pharmacological strategies for the prevention and alleviation of sleep disorders. Existing evidence consistently demonstrates that exercise therapy safely and effectively improves physical performance and quality of life among hospitalized patients with cancer and hematologic malignancies. de Souza et al. (46) demonstrated that low-to-moderate intensity combined aerobic and resistance training significantly preserves physical performance scores in patients undergoing hematopoietic stem cell transplantation, with no reported exercise-related adverse events (46). Borsati et al. (47) further demonstrated that exercise interventions significantly improve health-related quality of life among patients with hematologic malignancies (47). These findings provide evidence-based support for the implementation of individualized exercise therapy within inpatient settings.

Integrating bibliometric evidence with the intellectual content of highly cited core publications reveals several mechanistically coherent translational pathways that extend beyond descriptive trend mapping. First, the methodological evolution within this domain—from polysomnography and actigraphy to Mendelian randomization and wearable-based digital trials—reflects a progressive refinement in causal inference capability. The emergence of Mendelian randomization studies during 2024–2025 is particularly consequential: by leveraging genetic instrumental variables, these studies disentangled the bidirectional causality between physical activity and insomnia that has constrained observational research for decades. The convergence of Mendelian randomization evidence demonstrating protective effects of physical activity on leukocyte telomere length and post-stroke functional recovery suggests that exercise functions as an upstream modulator of biological aging processes underpinning sleep regulation, rather than merely serving as a symptomatic sleep aid. Second, intervention studies indexed within the high-centrality “sleep quality–actigraphy–college students” thematic cluster demonstrate that the efficacy of exercise interventions is neither universal nor mechanistically uniform. Moderate-intensity aerobic exercise and yoga improve sleep efficiency through distinct pathways: the former primarily enhances slow-wave sleep propensity via thermoregulatory and adenosinergic mechanisms, whereas the latter reduces pre-sleep cognitive arousal through parasympathetic activation. The identification of mobile phone addiction and sedentary behavior as negative moderators in this population indicates that digital-age lifestyle factors may override the sleep-promoting effects of exercise, necessitating multi-component interventions that simultaneously target activity promotion and screen-time reduction. Third, population stratification across thematic clusters reveals that the physical activity–insomnia relationship is moderated by life-stage and disease context in ways that challenge one-size-fits-all prescriptions. For cancer survivors, fatigue operates as a mediating mechanism linking exercise to quality-of-life improvements, suggesting that sleep benefits in this population may be secondary to central fatigue reduction rather than direct sleep architecture modification. For older adults, the U-shaped association between sleep duration and insomnia risk evident across multiple longitudinal cohorts implies that exercise recommendations must be calibrated to prevent both insufficient and excessive sleep, with 7–8 hours emerging as a non-pharmacological target. For hematologic malignancy inpatients, the safety and efficacy of low-to-moderate intensity exercise during hospitalization indicate that even acute care settings represent viable implementation contexts, although sleep-specific mechanisms (e.g., circadian entrainment versus anxiolysis) remain underexplored. Collectively, these observations suggest that the physical activity–insomnia field is transitioning from associative description to precision intervention, wherein bibliometrically identified thematic clusters can guide hypothesis generation for mechanism-driven randomized controlled trials. The “genetic causality–digital intervention” era heralded in our 2024–2025 analysis portends a future in which wearable-derived phenotypes, polygenic risk scores, and artificial intelligence-enabled exercise prescriptions are integrated into closed-loop sleep health systems. Realizing this vision will require transdisciplinary collaboration spanning sport science, sleep medicine, genetic epidemiology, and digital health engineering—themes that our centrality–density analysis confirms are already converging in the intellectual core of this field.

Cross-database validation between Scopus and Web of Science demonstrates strong convergent validity for the identified research trends in the PA-insomnia field. Despite inherent scope differences—WoS emphasizing high-impact disciplinary journals with stringent selection criteria versus Scopus adopting broader multidisciplinary coverage—the two databases exhibited approximately 85%–90% concordance across keyword co-occurrence patterns, thematic evolution trajectories, and temporal publication trends. Both datasets independently corroborated the four-stage paradigmatic shift from clinical disease-oriented research toward a multidimensional integrative model encompassing sleep, psychological, behavioral, and methodological dimensions. Notably, database-specific divergences were primarily confined to disciplinary emphases rather than substantive thematic discrepancies: Scopus leaned toward clinical medicine with greater representation of disease-specific and neurobiological terms, whereas Web of Science foregrounded methodological rigor and population-specific public health applications. These findings collectively indicate that our primary results are robust against database-specific biases and reflect genuine field-level developments rather than coverage artifacts. The high cross-database consistency thus strengthens confidence in the reliability and generalizability of the identified research evolution patterns, supporting their validity for informing future research prioritization and interdisciplinary collaboration in the PA-insomnia domain.

## 5 Strengths and limitations

This study leverages a comprehensive dataset of 2,224 English-language core articles from the Web of Science Core Collection (2010–2025), spanning a 16-year period—both the temporal scope and sample size substantially exceed those of previous reviews in this domain. Methodological rigor was enhanced through triangulation across three mainstream bibliometric tools (R-bibliometrix, Biblioshiny, and VOSviewer) and synchronization of four analytical visualizations (co-occurrence networks, bibliographic coupling, overlay visualizations, and strategic quadrants), revealing the dynamic migration of motor, specialized, basic, and emerging themes and providing an actionable priority roadmap for future research. Critically, cross-database validation with an independent Scopus dataset (4,754 records) using conceptually equivalent search strategies demonstrated approximately 85%–90% concordance across keyword co-occurrence patterns, thematic evolution trajectories, and temporal publication trends, with both databases independently corroborating the four-stage paradigmatic shift. Database-specific divergences were confined to disciplinary emphases (Scopus leaning toward clinical medicine; Web of Science foregrounding methodological and public health perspectives) rather than substantive thematic discrepancies, indicating that our results reflect genuine field-level developments rather than coverage artifacts. Furthermore, based on the concurrent rise of genetic causal evidence and digital intervention RCTs, we propose a scalable, replicable “sleep hygiene plus exercise package” as a precision public health pathway. Limitations include restriction to English-language databases, potentially overrepresenting Western research output while underrepresenting non-Western contributions; potential subjective bias in keyword consolidation and temporal classification; the descriptive rather than causal nature of bibliometric findings; and the use of Scopus data primarily for trend verification rather than full analytical replication.

## 6 Conclusion

Based on bibliometric and knowledge-mapping analyses of 2,224 publications from the Web of Science Core Collection (2010–2025), this study provides the first global-scale systematic confirmation that the physical activity–insomnia domain has bibliometric indicators suggest a four-stage thematic evolution pattern: from metabolic association validation through pandemic stress responsiveness and preventive intervention integration to genetic causal precision. China and the United States collectively dominate the research network with a combined output share of 41.4%, with the Harvard system, University of California system, and the Sleep Medicine journal portfolio constituting the primary knowledge production bases. Mendelian randomization, actigraphy, and digital randomized controlled trials have supplanted traditional cross-sectional surveys as the new methodological “golden triad.” Populations including university students, cancer survivors, and children with long COVID—characterized by high external validity—are increasingly employed to empirically test causal pathways identified through genetic instrumental variables. Sleep quality, fatigue, and quality of life, as key endpoints amenable to concurrent modification through exercise intervention, exhibit the highest betweenness centrality and internal density within the 122 high-frequency keyword network. Toward 2030, the field urgently requires: (1) transcontinental, multi-center, open-access genetic-behavioral cohorts utilizing nested Mendelian randomization–RCT designs to precisely quantify causal effect sizes of physical activity across distinct insomnia phenotypes; (2) artificial intelligence-enabled wearable-based real-time sleep-emotion-metabolism digital twin systems to enable dynamic optimization of individualized exercise prescriptions; and (3) integration of multi-component “sleep hygiene plus exercise” interventions into oncology and chronic disease management guidelines, utilizing fatigue as a mediator to establish replicable, scalable, and cost-effective pathways for quality-of-life enhancement. Only through these concerted efforts can existing evidence be translated into precise, equitable, and sustainable sleep health practices within the context of escalating global aging and chronic disease burdens, thereby achieving the dual advancement of brain health and quality of life. Furthermore, cross-database validation substantiated the robustness of these findings and trends.

## Data Availability

The original contributions presented in the study are included in the article/**Supplementary Material**. Further inquiries can be directed to the corresponding author.
