# Supplementary material for: Research advances in physical activity and insomnia: a bibliometric and thematic evolution analysis
Source: Front Psychiatry. 2026 Jun 10;17:1801508. doi: 10.3389/fpsyt.2026.1801508 (PMC13291116; doi:10.3389/fpsyt.2026.1801508)
Supplement: Supplementary file 1 [file Table1.docx]

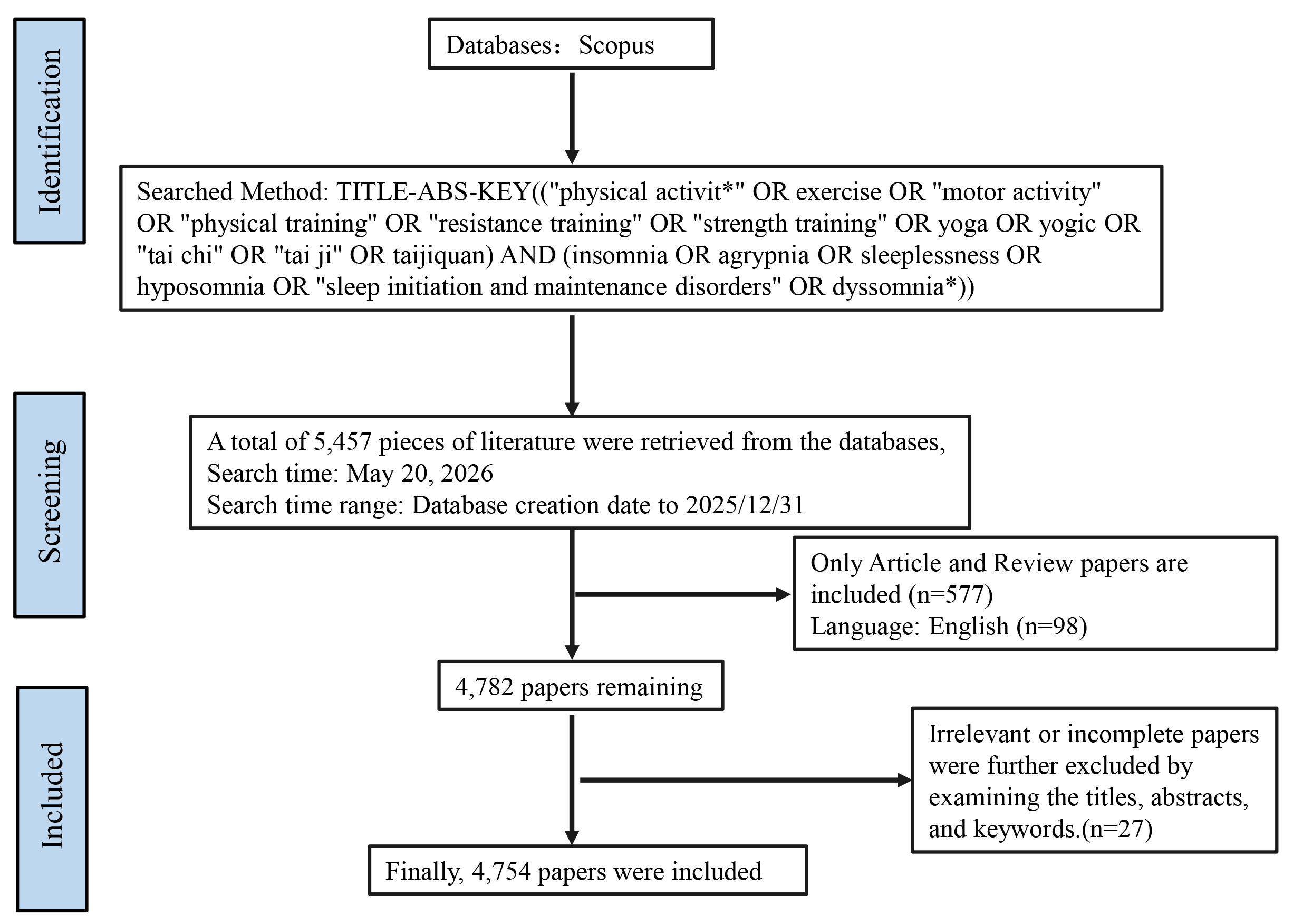


FIGURE S1. Flow diagram for the screening.


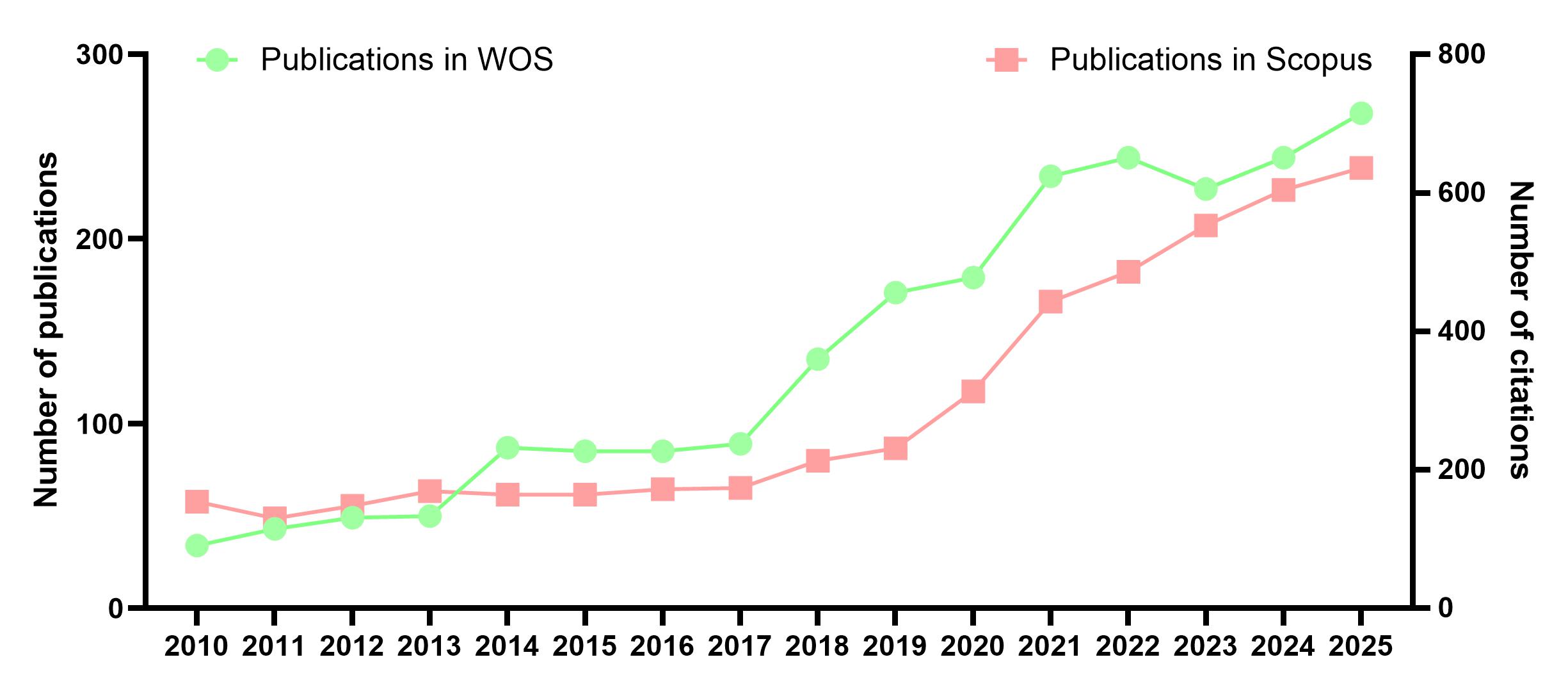


FIGURE S2. The annual publication trend of the database.


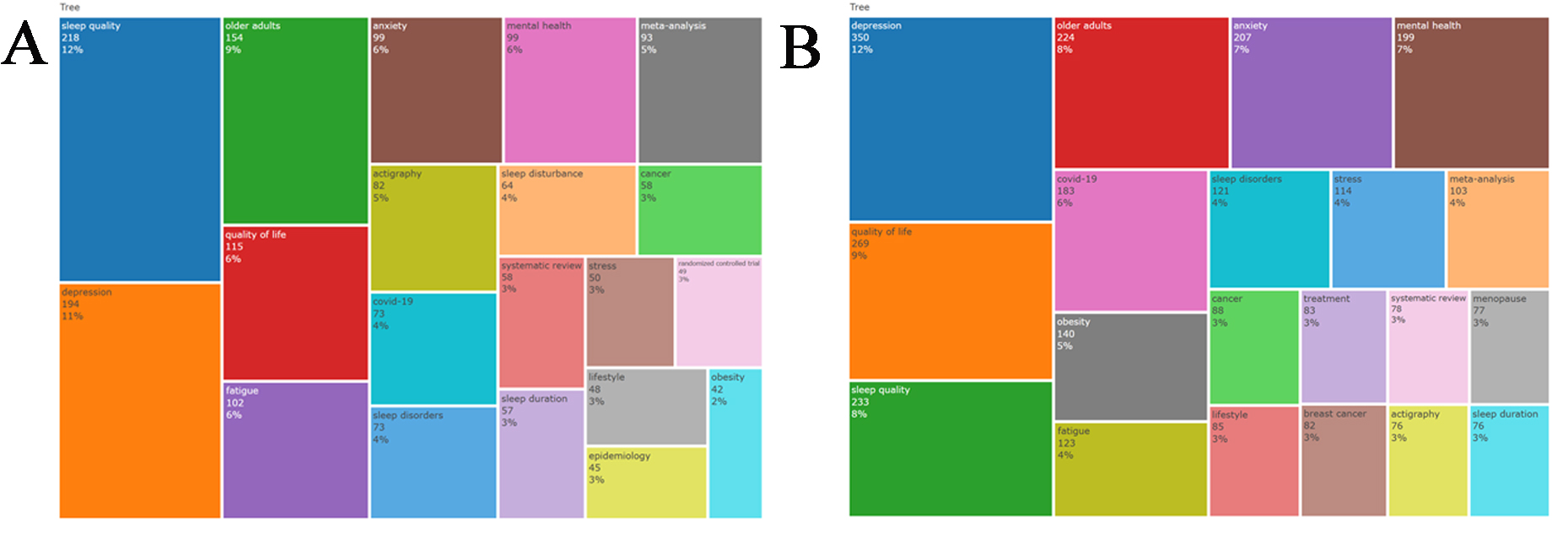


FIGURE S3. Tree map of the most frequent author keywords. (A)Web of Science database keywords. (B)Scopus Database Keywords.


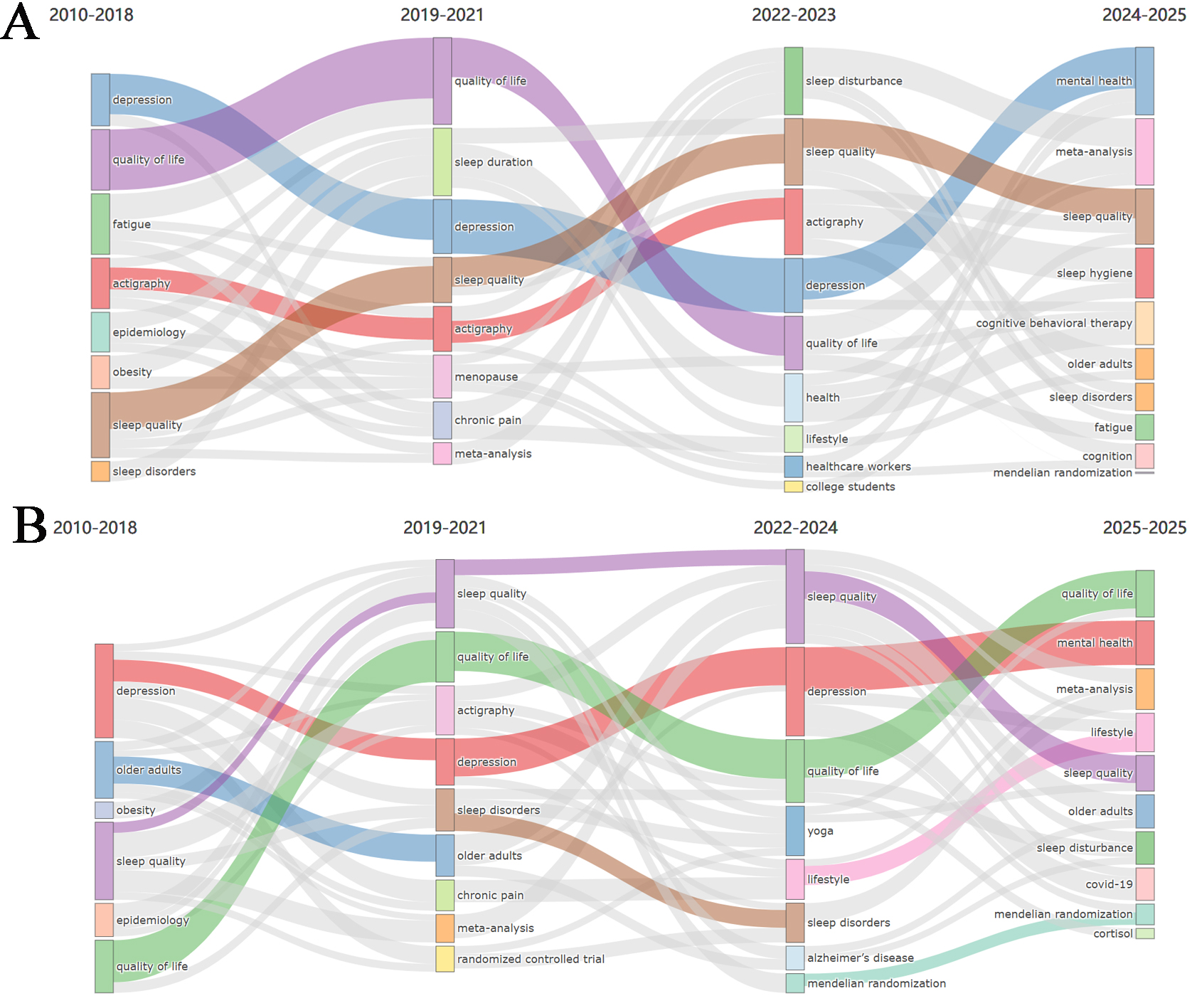


FIGURE S4. Analysis of Theme Evolution. (A) Evolution of Topics in Web of Science Database. (B) Evolution of Topics in Scopus Database.
